# Amylo-AFFECT-QOL, a self-reported questionnaire to assess health-related quality of life and to determine the prognosis in cardiac amyloidosis

**DOI:** 10.3389/fcvm.2023.1124660

**Published:** 2023-03-14

**Authors:** Mounira Kharoubi, Mélanie Bézard, Amaury Broussier, Arnault Galat, Romain Gounot, Elsa Poullot, Valérie Molinier-Frenkel, Pascale Fanen, Benoit Funalot, Emmanuel Itti, François Lemonnier, Gagan Deep Sing Chadha, Soulef Guendouz, Sophie Mallet, Amira Zaroui, Vincent Audard, Etienne Audureau, Philippe Le Corvoisier, Luc Hittinger, Violaine Planté Bordeneuve, Jean-Pascal Lefaucheur, Aurélien Amiot, Emilie Bequignon, Sophie Bartier, Vincent Leroy, Emmanuel Teiger, Silvia Oghina, Thibaud Damy

**Affiliations:** ^1^AP-HP (Assistance Publique-Hôpitaux de Paris), Department of Cardiology, Henri Mondor University Hospital, Créteil, France; ^2^AP-HP (Assistance Publique-Hôpitaux de Paris), French National Referral Centre for Cardiac Amyloidosis, Cardiogen Network, Henri Mondor University Hospital, Créteil, France; ^3^AP-HP (Assistance Publique-Hôpitaux de Paris), GRC Amyloid Research Institute, Henri Mondor University Hospital, Créteil, France; ^4^AP-HP (Assistance Publique-Hôpitaux de Paris), DHU A-TVB, Henri Mondor University Hospital, Créteil, France; ^5^Université Paris-Est Créteil (UPEC), Creteil, France; ^6^AP-HP (Assistance Publique-Hôpitaux de Paris), Hopitaux Henri-Mondor/Emile Roux, Department of Geriatrics, Limeil-Brévannes, France; ^7^AP-HP (Assistance Publique-Hôpitaux de Paris), Lymphoid Malignancies, Henri Mondor University Hospital, Créteil, France; ^8^AP-HP (Assistance Publique-Hôpitaux de Paris), Department of Pathology, Henri Mondor University Hospital, Créteil, France; ^9^University Paris Est Créteil, Institut National de la Santé et de la Recherche Médicale (INSERM) U955, Institut Mondor de Recherche Biomédicale (IMRB), Créteil, France; ^10^AP-HP (Assistance Publique-Hôpitaux de Paris), Département de Génétique, Hôpital Universitaire Henri Mondor, Créteil, France; ^11^AP-HP (Assistance Publique-Hôpitaux de Paris), Department of Nuclear Medicine, Henri Mondor University Hospital, Créteil, France; ^12^AP-HP (Assistance Publique-Hôpitaux de Paris), Nephrology and Renal Transplantation Department, Henri Mondor Hospital University, Centre de Référence Maladie Rare « Syndrome Néphrotique Idiopathique », Fédération Hospitalo-Universitaire « Innovative Therapy for Immune Disorders », Créteil, France; ^13^AP-HP (Assistance Publique-Hôpitaux de Paris), Public Health Department, Henri Mondor University Hospital, Créteil, France; ^14^INSERM Clinical Investigation Centre 1430, AP-HP (Assistance Publique-Hôpitaux de Paris), Henri Mondor University Hospital, Créteil, France; ^15^AP-HP (Assistance Publique-Hôpitaux de Paris), Department of Neurology, Henri Mondor University Hospital, Créteil, France; ^16^AP-HP (Assistance Publique-Hôpitaux de Paris), Department of Neurophysiology, Henri Mondor University Hospital, Créteil, France; ^17^AP-HP (Assistance Publique-Hôpitaux de Paris), Hepato Gastro Enterology Department, Henri Mondor University Hospital, Créteil, France; ^18^AP-HP (Assistance Publique-Hôpitaux de Paris), Department of Otolaryngology, Henri Mondor University Hospital, Créteil, France

**Keywords:** cardiac amyloidosis, quality of life, prognosis, transthyretin, self-reported questionnaire

## Abstract

**Background and aims:**

Self-reported questionnaires are useful for estimating the health-related quality of life (HR-QoL), impact of interventions, and prognosis. To our knowledge, no HR-QoL questionnaire has been developed for cardiac amyloidosis (CA). This study aimed to validate Amylo-AFFECT-QOL questionnaire to assess HR-QoL and its prognostic value in CA.

**Methods:**

A self-reported questionnaire, “Amylo-AFFECT” had been designed and validated for CA symptoms evaluation and screening by physicians. It was adapted here to assess HR-QoL (Amylo-AFFECT-QOL) and its prognostic value in CA. To validate the theoretical model, internal consistency and convergent validity were assessed, particularly correlations between Amylo-AFFECT-QOL and the HR-QoL Minnesota Living Heart Failure (MLHF) questionnaire.

**Results:**

Amylo-AFFECT-QOL was completed by 515 patients, 425 of whom (82.5%) had CA. Wild-type and hereditary transthyretin amyloidosis (ATTRwt and ATTRv) and immunoglobulin light-chain amyloidosis (AL) were diagnosed in 47.8, 14.7, and 18.8% of cases, respectively. The best HR-QoL evaluation was obtained with five dimensions: “Heart failure,” “Vascular dysautonomia,” “Neuropathy,” “Ear, gastrointestinal, and urinary dysautonomia,” and “Skin or mucosal involvement.” The global Amylo-AFFECT-QOL and MLHF scores showed significant positive correlations (rs = 0.72, *p* < 0.05). Patients with a final diagnosis of CA had a global Amylo-AFFECT-QOL score significantly higher than the control group composed by patients with other diagnoses (22.2 ± 13.6 vs. 16.2 ± 13.8, respectively, *p*-value < 0.01). According to the Amylo-AFFECT-QOL global results, ATTRv patients’ QoL was more affected than AL patients’ QoL or ATTRwt patients’ QoL. Patients with a higher HR-QoL score had a greater risk of death or heart transplant after 1 year of follow-up (log-rank < 0.01).

**Conclusion:**

Amylo-AFFECT-QOL demonstrates good psychometric properties and is useful for quantifying HR-QoL and estimating CA prognosis. Its use may help to improve overall management of patients with CA.

## 1. Introduction

Amyloidosis is a severe, progressive, and fatal systemic disease characterized by the accumulation of insoluble fibrillar proteins in the extracellular matrix of various organs including the heart and peripheral nerves (1). There are three main types of cardiac amyloidosis (CA): immunoglobulin light-chain (AL) amyloidosis, due to amyloidogenic monoclonal light-chain production by a plasma cell clone, hereditary transthyretin amyloidosis (ATTRv) caused by the deposition of mutated transthyretin (TTR), and wild-type (non-hereditary) TTR amyloidosis (ATTRwt) (2–5). The potential for amyloid deposits to affect almost any organ system and the wide range of clinical expression (6) explain the major impact of the symptoms of amyloidosis on the daily life of patients.

Nowadays, health-related quality of life (HR-QoL) is considered an important dimension in the management of patients and is often a surrogate endpoint in trials testing TTR stabilizers or RNA interference drugs (7, 8). Structured assessment of HR-QoL is considered important in promoting patient-centric care, placing the patient’s perspective at the forefront to identify areas of specific need and guide management of the disease. It also provides a framework for clinical monitoring. Several validated generic HR-QoL assessment instruments have been developed to date in Heart Failure such as The Minnesota Living with Heart Failure or the Kansas City Cardiomyopathy Questionnaire (7, 9) but not, to our knowledge, in cardiac amyloidosis. A reduction in HR-QoL has been shown to be an independent predictor of increased hospitalization and mortality on heart failure (10).

Currently, amyloidosis patients’ HR-QoL has been assessed in trials or research studies using different generic questionnaires such as EQ-5D (11) or SF-36 with or without disease-specific questionnaires such as Norfolk QoL-DN (12–14), an instrument to assess QoL in diabetic polyneuropathy; Hematology Patient Reported Symptom Screen (HPRSS) which is a three point questionnaire on fatigue, pain, and overall QOL (15). The Kansas City Cardiomyopathy Questionnaire (KCCQ) (16) specific to heart failure or Minnesota Living Heart Failure (MLHF) Questionnaire (17) also specific to heart failure but more generic than KCCQ. However, none of them is specific to CA and most evaluate only one organ. In this multisystemic disease, there is clearly a need for a self-reported questionnaire that measures the impact of multiple organ involvement on QoL.

To the best of our knowledge, Amylo-AFFECT is the first self-symptoms-reported questionnaire created especially for CA (in press). It is easy to use, and its generalist design allows screening of all CA symptoms and helps physicians in making a diagnosis (in press). We assumed that, with some modifications, Amylo-AFFECT could be also relevant to evaluate HR-QoL of CA patients.

The aim of this study was to validate a specific self-reported quality of life questionnaire, “Amylo-AFFECT-QOL” to assess the QoL and its prognostic value in CA patients.

## 2. Materials and methods

### 2.1. Development of Amylo-AFFECT-QOL as a self-reported QOL questionnaire in patients with suspicion of cardiac amyloidosis

The Amylo-AFFECT was developed in 2014 by the French National Referral Center. This questionnaire was designed as a checklist for all the symptoms that could be associated with CA. The selection of questions was conducted by a multidisciplinary team to ensure the scientific and clinical relevance of the process in the amyloidosis monitoring network, including cardiologists, hematologists, nephrologists, neurologists, and experts in questionnaire conception in association with CA patients.

The first stage included the creation of a verbatim report based on a review of the literature and qualitative information collected during semi-structured and unstructured interviews with CA patients to discuss their complaints and distress related to the disease until saturation. As described previously, based on reports and inputs from a multidisciplinary working group, the major identified concerns were the geographical origin, sex and age, orthostatic hypotension, neuromuscular, carpal tunnel, digestive symptoms, urinary or genital disorders, and their impact on the skin, nails, and thyroid. Thus, Amylo-AFFECT is composed of a set of 34 questions produced and grouped according to their content. After the test, some questions concerning sexual problems and thyroid disorders were removed because they were irrelevant. To measure symptoms, patients were asked about the presence of symptoms (yes or no) and when the symptom occurred (last 2 years or more than 2 years ago).

Regarding the creation of Amylo-AFFECT-QOL self-reported questionnaire, the scientific committee decided to select the same items to measure discomfort using a Likert scale defined as follows: 0 no discomfort, 1 mild discomfort, 2 moderate discomfort, 3 severe discomfort). Only Likert scale scores were used to assess patients’ QoL.

A cross-sectional survey was conducted with > 100 adult patients with medically diagnosed CA. The French version of the questionnaire was pretested with the first 10 patients to evaluate comprehensibility, ambiguity, misunderstanding, and acceptability, and changes were made based on their comments. To be eligible, patients had to be able to read, understand, and speak French and lack any cognitive impairment. Psychometric properties of the scale were assessed. A global score was calculated by summing the individual item scores, with a higher global score representing a higher symptom burden. The translation and cross-cultural assessment methods are detailed in Supplementary material 2.

### 2.2. Study population

**FIGURE 1:**
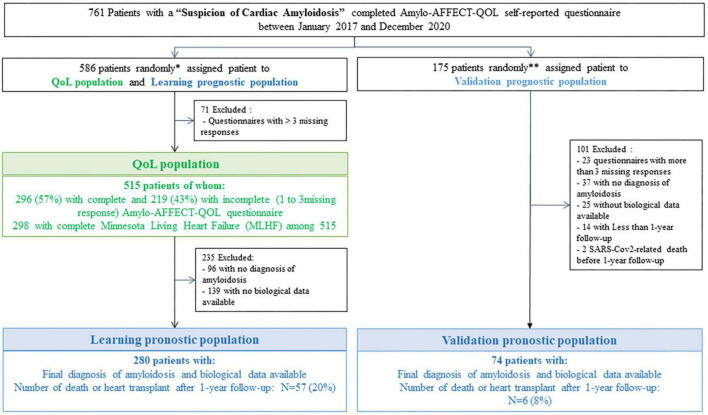
Flowchart of study populations: QoL population and learning and validation population. *Included all patients who completed Amylo-AFFECT questionnaire between January 2017 and January 2019 and a randomly sample of patients who completed Amylo-AFFECT questionnaire between January 2019 and December 2019. ^**^Included a randomly sample of patients who completed Amylo-AFFECT questionnaire between January 2019 and December 2019 and all patients who completed Amylo-AFFECT questionnaire between January 2020 and December 2020.

The cohort study was prospectively conducted in France by the French National Referral Center between January 2017 and December 2020. At their first visit to the Center, patients referred for suspicion of CA were asked to complete the Amylo-AFFECT-QOL and the MLHF questionnaire (Figure 1), which was selected for its large-scale screening of potential damage in case of heart failure compared to that of the other questionnaires available. Suspected CA was defined as one or more signs of heart failure: a hospital admission for heart failure in the previous 12 months, treatment with a diuretic, clinical signs of heart failure (leg oedema or elevated jugular venous pressure), and diastolic dysfunction on echocardiography. All questionnaires were completed by patients at time when the diagnosis was not yet confirmed and for both AL and ATTR patients, none of them had been treated before and any patients had been before arriving for suspicion of cardiac amyloidosis to the Center.

Patients with a final diagnostic of cardiopathy and those who fulfilled the Amylo-AFFECT-QOL questionnaire were included in the study between January 2017 and December 2020.

Populations analyses were distributed in three groups as follow (Figure 1): The first random population of patients referred for “Suspicion of CA” who had filled the Amylo-AFFECT-QOL was used as the base for HR-QoL evaluation (QoL population). From this first random group, the Learning prognostic population for prognostic model determination was selected after excluding patients without amyloidosis diagnosis and those without biological data available. A second random population was identified as the Validation prognostic population after exclusion of patients without amyloidosis and without available biological data and was used to validate the prognostic models.

### 2.3. QoL evaluation and prognostic values of Amylo-AFFECT-QOL questionnaire

#### 2.3.1. Amylo-AFFECT-QOL determination value using the QoL population

The QoL population described in the study population paragraph was used to determine the QoL value using the Amylo-AFFECT-QOL questionnaire part. Psychometric analysis included the assessment of item characteristics, construction and validation of the theoretical model, internal consistency, and convergent validity.

#### 2.3.2. Answers description

Descriptive analyses were performed to study the distribution of individual items and global scores, inform on acceptability (% missing values), and identify potential ceiling and/or floor effects when a majority of item responses were distributed at either end of the scale. A simple hot-deck imputation was performed to impute the missing data for subsequent analyses.

#### 2.3.3. Correlation between questions

The Spearman’s rank correlation coefficient matrix (rs) was computed to identify whether highly correlated items should be omitted for redundancy (inter-item correlation, rs > 0.8). A correlation network plot was constructed from these results to graphically illustrate the relationships.

#### 2.3.4. Construction of a theoretical model

The construction of the theoretical model (factor structure) of the Amylo-AFFECT-QOL questionnaire was assessed through exploratory factor analysis (principal factor method) to examine the underlying constructs and characterize the scale dimensionality. We assumed that highly interrelated items contained similar information and were grouped under a common factor. Five dimensions are determined.

#### 2.3.5. Validation of the theoretical model

Internal consistency reliability was assessed using a confirmatory factorial analysis. A theoretical model was defined within an exploratory factorial analysis, during which variables of the Amylo-AFFECT-QOL questionnaire were associated with the five dimensions, and a confirmatory factorial analysis was used to determine the extent to which this theoretical model correctly reproduced the collected data. Several fit indices were calculated to assess internal consistency (Supplementary Material 2). Internal consistency reliability (homogeneity of the items) was assessed by calculating the Cronbach’s alpha. A coefficient score of > 0.8 indicated good internal consistency.

#### 2.3.6. Convergent validity

Convergent validity was studied by assessing correlations between the Amylo-AFFECT-QOL total score and dimensions score (total score and dimension scores were calculated using the sum of the scores of each item composing the dimension) and the HR-QoL MLHF (details in Supplementary material 2), a validated 21-item questionnaire for assessing HR-QoL in patients with heart failure (18). Spearman’s rank correlation coefficients were computed between the Amylo-AFFECT-QOL score and each MLHF domain score, namely physical (8 items, score 0–40), emotional (5 items, 0–25), and other items (8 items, 0–40). Convergent validity measured the relation between the Amylo-AFFECT-QOL scores and the scores of other scales that measured similar but not strictly equivalent constructs. Coefficients < 0.3 were considered as weak, 0.3–0.5 as moderate, and >0.5 as strong.

### 2.4. Assessment of Amylo-AFFECT-QOL prognostic value using the learning prognostic population and the validation prognostic population

As described below, the Learning prognostic population, including patients with amyloidosis diagnosis and the completed Amylo-AFFECT-QOL questionnaire, was used to construct the prognostic model.

A hot-deck multiple imputation (*N* = 5 imputations) was performed to impute missing data for the prognostic study [all results were pooled using the Rubin formula (19)]. Three logistic regression models were developed using the Learning prognostic population according to the following inputs: Amylo-AFFECT-QOL dimension scores alone, biological markers (troponin and NT-proBNP) alone, or both. The output variable was defined as death or heart transplant after 1 year of follow-up. The probability of this output variable was calculated from the parameters obtained with the logistic regression according to the formula written below, where X_*i*_ refers to the input (Amylo-AFFECT-QOL dimensions or biological markers), and β_*i*_ is the parameter obtained with the logistic regression.


$$P\,r\,\left(O\,u\,t\,p\,u\,t/X_{i}\right)=\frac{\exp\left(\sum_{i}\beta_{i}\,X_{i}\right)}{1+\exp\left(\sum_{i}\beta_{i}\,X_{i}\right)}$$

This probability was used as the prognostic score. Receiver operating characteristic (ROC) curves were established, and the area under the curve (AUC) was calculated for each model and compared. For a given prognostic score, the sensitivity, specificity, accuracy, and positive and negative predictive values were assessed for the three models.

Kaplan–Meier survival analysis using the log-rank test was performed to describe the link between death or heart transplant and the calculated prognostic value for each model: Amylo-AFFECT-QOL scores alone, biological markers alone, and Amylo-AFFECT-QOL scores and associated biological markers. This model was tested and confirmed using the Validation prognostic population, an external group of patients with a diagnosis of amyloidosis and a completed Amylo-AFFECT-QOL questionnaire.

### 2.5. Statistical analyses

All statistical analyses were performed using Statistical Analysis Software version 9.4 (SAS Institute, Inc., Créteil, France). The study was approved by the ethics committee (authorization number #1431858), and informed consent for participation in this research was obtained from all patients. Data were recorded electronically in the Henri Mondor Amyloidosis Network registry, as authorized by the French CNIL (Commission National de l’Informatique et des Libertés). All data were centralized in a secure database.

## 3. Results

### 3.1. Population characteristics

As described in Figure 1, the HR-QoL assessment of the Amylo-AFFECT-QOL questionnaire was based on the QoL population composed of 515 patients with a mean age of 73.5 ± 11.4 years. The majority were men (74.6%) and Caucasian (69.3%), and 433 (84.1%) had a diagnosis of amyloidosis; among them, 425 (82.5%) had cardiac involvement (Table 1). A diagnosis of ATTRwt was made in 47.8% of cases, ATTRv with cardiopathy with or without neuropathy in 14.7%, and AL in 18.8% of cases. The main characteristics of the sample, including sociodemographic characteristics, biological features, history, cardiovascular characteristics, risk factors, and echocardiographic characteristics, are shown in Table 1. Among the 515 patients, 298 completed the MLHF questionnaire (Figure 1).

**TABLE 1:** Characteristics of the QoL population used for the assessment of quality of life, according to the type of amyloidosis: immunoglobulin light-chain amyloidosis (AL) and wild type and hereditary amyloidosis (ATTRwt and ATTRv).

	QoL population					
Characteristics	Missing value	AL	ATTRwt	ATTRv	Others*	Overall
		*N* = 97	*N* = 246	*N* = 76	*N* = 96	*N* = 515
**Clinical characteristics**						
Male, n (%)	0	63 (64.9%)	208 (84.6%)	50 (65.8%)	63 (66.3%)	384 (74.6%)
Age, years, mean ± SD	0	67.8 (±9.6)	79.4 (±7.0)	72.7 (±9.7)	64.6 (±14.1)	73.5 (±11.3)
BMI, kg/m^2^, mean ± SD	72	24.2 (±6.1)	25.4 (±3.9)	24.4 (±3.7)	26.2 (±4.5)	25.2 (±4.5)
Ethnic group, n (%)						
Caucasian	0	70 (72.2%)	217 (88.2%)	20 (26.7%)	49 (51.0%)	356 (69.1%)
Sub-Saharan Africa		3 (3.1%)	1 (0.4%)	26 (34.2%)	13 (13.5%)	43 (8.3%)
Afro-Caribbean		6 (6.2%)	6 (2.4%)	18 (23.7%)	12 (12.5%)	42 (8.2%)
North Africa		8 (8.2%)	10 (4.1%)	8 (10.5%)	11 (11.5%)	37 (7.2%)
Portuguese		6 (6.2%)	9 (3.7%)	1 (1.3%)	8 (8.43)	24 (4.7%)
South America, Asian, Other		4 (4.1%)	3 (1.2%)	3 (3.9%)	3 (3.1%)	13 (2.5%)
Amyloidosis, n (%)	0	97 (100%)	246 (100%)	76 (100%)	14 (14.6%)	433 (84.1%)
Cardiac amyloidosis, n (%)	0	97 (100%)	246 (100%)	76 (100%)	6 (6.3%) **	425 (82.5%)
**CV characteristics**						
NYHA class I–II vs. III–IV, n (%) I–II	50	53 (54.6%)	153 (62.2%)	42 (55.2%)	59 (61.4%)	307 (59.6%)
III–IV		37 (38.1%)	77 (31.3%)	30 (38.5%)	14 (14.6%)	158 (30.7%)
Heart rate, beats/min, mean ± SD	5	82.0 (±15.6)	75.3 (±14.0)	76.4 (±15.4)	74.2 (±13.9)	76.5 (±14.7)
Systolic blood pressure, mmHg, mean ± SD	19	116.8 (±21.3)	131.2 (±19.1)	126.0 (±22.0)	138.3 (±20.4)	129.1 (±21.3)
Diastolic blood pressure, mmHg, mean ± SD	19	71.4 (±11.8)	75.7 (±11.8)	75.7 (±13.8)	77.8 (±13.8)	75.3 (±12.6)
Atrial fibrillation, n (%)	60	11 (12.8%)	78 (35.1%)	13 (19.4%)	11 (13.8%)	113 (24.8%)
Pacemaker, n (%)	0	14 (14.4%)	72 (29.3%)	17 (22.4%)	10 (10.4%)	113 (21.9%)
ICD, n (%)	0	14 (14.4%)	72 (29.3%)	17 (22.4%)	10 (10.4%)	113 (21.9%)
**History**						
Carpal tunnel surgery or symptoms, n (%)	62	33 (37.1%)	165 (75.3%)	60 (82.2%)	11 (15.3%)	269 (59.4%)
Deafness, n (%)	95	24 (28.9%)	153 (72.9%)	28 (40.6%)	18 (31.0%)	223 (53.1%)
**CV risk factors**						
Diabetes, n (%)	0	15 (15.5%)	39 (15.9%)	13 (17.1%)	16 (16.7%)	83 (16.1%)
Dyslipidemia, n (%)	0	22 (22.7%)	92 (37.4%)	19 (25.0%)	18 (18.8%)	151 (29.3%)
Hypertension, n (%)	0	35 (36.1%)	144 (58.5%)	46 (60.5%)	42 (43.8%)	267 (51.8%)
**Biology**						
Nt-proBNP, pg/mL: median [iqr]	46	4005.0 [1266.5–8987.0]	2547.0 [1318.0–5282.0]	2202.0 [1023.0–4159.0]	808.0 [139.0–2441.0]	2209.0 [960.0–5160.0]
Nt-proBNP = 600, pg/mL* n (%)	46	77 (87.5%)	206 (93.2%)	56 (81.2%)	54 (59.3%)	393 (83.8%)
Nt-proBNP-eGFR Staging n (%):	122					
Stage 1		22 (32.8%)	99 (54.1%)	41 (59.4%)	51 (68.9%)	213 (54.2%)
Stage 2		28 (41.8%)	56 (30.6%)	19 (27.5%)	14 (18.9%)	117 (29.8%)
Stage 3		17 (25.4%)	28 (15.3%)	9 (13.0%)	9 (12.2%)	63 (16.0%)
Troponin T HS, ng/mL: median [iqr]	78	83.0 [44.5–148.5]	62.0 [42.0–85.0]	67.0 [37.0–110.0]	22.0 [10.0–43.0]	59.0 [31.0–90.0]
Hemoglobin, g/dL, mean ± SD	45	12.6 (±1.9)	13.5 (±1.6)	12.7 (±1.7)	12.9 (±2.1)	13.1 (±1.8)
Creatinine, μmol/L, mean ± SD	16	137.9 (±91.9)	114.5 (±43.2)	115.9 (±50.2)	150.6 (±180.7)	125.9 (±95.6)
eGFR, ml/min/1.73m^2^, mean ± SD	88	59.3 (±29.2)	61.5 (±19.1)	69.0 (±28.8)	64.4 (±30.2)	62.9 (±25.1)
**Echocardiography characteristics**						
LVEF, %, mean ± SD	62	54.6 (±9.6)	50.4 (±11.3)	49.8 (±13.7)	55.3 (±12.3)	51.9 (±11.8)
IVST, mm, mean ± SD	51	15.8 (±3.4)	17.6 (±3.3)	17.3 (±3.6)	13.6 (±3.3)	16.6 (±3.7)
GL Strain, %, mean ± SD	75	11.5 (±4.3)	10.8 (±3.6)	11.5 (±3.8)	14.7 (±4.6)	11.7 (±4.1)

*“Other” include not typed amyloidosis, ATTRv with neuropathy only, Amyloidosis non-AL and non-ATTR, localized amyloidosis, Carrier, hematopathy other than amyloidosis, and cardiopathy other than amyloidosis.

**Cardiac amyloidosis includes 4 cases (4.2%) ATTRv with neuropathy only, and 2 cases of no typed amyloidosis (2.1%).

SD, standard deviation; BMI, body mass index; CV, cardiovascular; NYHA, New York Heart Association; ICD, implantable cardioverter defibrillator; eGFR, estimated glomerular filtration rate; LVEF, left ventricular ejection fraction; IVST, interventricular septal thickness.

As described in Figure 1, among the 515 patients in the QoL population, a subgroup of 280 patients with available biological data and a final diagnosis of CA, constituting the learning prognostic population, was used to evaluate the prognostic value of the Amylo-AFFECT-QOL questionnaire. The mean age was 75.8 ± 9.7, the majority were men (76.4%) and Caucasian (70.7%). The median NT-proBNP and troponin levels were 2585.5 pg/mL [1364.5–5729.5] and 66.5 ng/mL [42.0–101.0], respectively (Table 2).

**TABLE 2:** Characteristics of (1) learning and (2) validation prognostic population used for the assessment of Amylo-AFFECT prognostic value, according to the type of amyloidosis: immunoglobulin light-chain amyloidosis (AL) and wild type and hereditary amyloidosis (ATTRwt and ATTRv).

	(1) Learning prognostic population					(2) Validation prognostic population				
Characteristics	Missing value	AL *N* = 64	ATTRwt *N* = 167	ATTRv *N* = 49	Overall *N* = 280	Missing value	AL *N* = 8	ATTRwt *N* = 55	ATTRv *N* = 11	Overall *N* = 74
**Clinical characteristics**										
Male, n (%)	0	42 (65.6)	142 (85.0)	30 (61.2)	214 (76.4)	0	5 (62.5)	51 (92.7)	9 (81.8)	65 (87.8)
Age, years, mean ± SD	0	67.4 (±9.8)	79.8 (±6.9)	72.8 (±9.8)	75.8 (±9.7)	0	70.2 (±10.3)	79.7 (±8.7)	74.9 (±10.6)	77.9 (±9.6)
IMC, kg/m^2^, mean ± SD	41	23.6 (±3.9)	25.6 (±4.1)	24.6 (±3.6)	25.0 (±4.1)	6	23.4 (±3.5)	25.7 (±3.8)	25.9 (±5.7)	25.5 (±4.1)
Ethnic group, n (%) Caucasian	0	43 (67.2)	143 (85.6)	12 (24.5)	198 (70.7)	0	7 (87.5)	51 (92.7)	2 (18.2)	60 (81.1)
Sub-Saharan Africa		3 (4.7)	1 (0.6)	16 (32.7)	20 (7.1)		0	0	3 (27.3)	3 (4.1)
Afro-Caribbean		4 (6.3)	5 (3.0)	14 (28.6)	23 (8.2)		0	0	4 (36.4)	4 (5.4)
North Africa		7 (10.9)	8 (4.8)	5 (10.2)	20 (7.1)		1 (12.5)	1 (1.8)	0	2 (2.7)
Portuguese		3 (4.7)	7 (4.2)	1 (2.0)	11 (3.9)		0	3 (5.5)	0	3 (4.1)
South America, Asian, Other		4 (6.3)	3 (1.8)	1 (2.0)	8 (2.9)		0	0	2 (18.2)	2 (2.7)
Amyloidosis, n (%)	0	64 (100)	167 (100)	49 (100)	280 (100)	0	8 (100)	55 (100)	11 (100)	74 (100)
Cardiac amyloidosis, n (%)	0	64 (100)	167 (100)	49 (100)	280 (100)	0	8 (100)	55 (100)	11 (100)	74 (100)
**CV characteristics**										
NYHA class I–II vs. III–IV, n (%) I–II	18	32 (50)	105 (62.9)	26 (53.1)	163 (58.2)	15	5 (62.5)	29 (52.7)	5 (45.4)	39 (52.7)
III–IV		28 (43.7)	51 (30.5)	20 (40.8)	99 (35.7)		3 (37.5)	15 (27.3)	2 (18.2)	20 (27.0)
Heart rate, beats/min, mean ± SD	2	82.1 (±15.5)	75.8 (±13.7)	78.1 (±16.4)	77.6 (±14.8)	11	96.6 (±28.1)	71.8 (±13.6)	73.8 (±12.1)	75.2 (±17.6)
Systolic blood pressure, mmHg, mean ± SD	11	116.5 (±23.0)	130.9 (±19.5)	126.0 (±24.2)	126.8 (±21.9)	11	106.9 (±18.0)	131.6 (±18.2)	105.3 (±40.2)	125.1 (±24.3)
Diastolic blood pressure, mmHg, mean ± SD	11	70.8 (±12.0)	75.7 (±12.2)	76.6 (±15.4)	74.8 (±12.9)	11	73.9 (±11.6)	74.0 (±13.1)	72.4 (±15.1)	73.8 (±13.0)
Atrial fibrillation, n (%)	35	7 (12.7)	52 (35.4)	9 (20.9)	68 (27.8)	6	1 (14.3)	17 (32.7)	1 (11.1)	19 (27.9)
Pacemaker, n (%)		8 (12.5)	50 (29.9)	9 (18.4)	67 (23.9)	0	2 (25.0)	23 (41.8)	2 (18.2)	27 (36.5)
ICD, n (%)	0	12 (18.8)	18 (10.8)	15 (30.6)	45 (16.1)	0	1 (12.5)	4 (7.3)	1 (9.1)	6 (8.1)
**History**										
Carpal tunnel surgery or symptoms, n (%)	27	21 (35.0)	103 (70.1)	38 (82.6)	162 (64.0)	65	0	4 (66.7)	0	4 (44.4)
Deafness, n (%)	44	13 (23.6)	102 (73.4)	16 (38.1)	131 (55.5)	66	0	2 (40.0)	0	2 (25.0)
**CV risk factors**										
Diabetes, n (%)	0	11 (17.2)	28 (16.8)	8 (16.3)	47 (16.8)	0	1 (12.5)	0	0	1 (1.4)
Dyslipidemia, n (%)	0	16 (25.0)	67 (40.1)	14 (28.6)	97 (34.6)	0	1 (12.5)	2 (3.6)	0	3 (4.1)
Hypertension, n (%)	0	28 (43.8)	99 (59.3)	32 (65.3)	159 (56.8)	0	1 (12.5)	3 (5.5)	0	4 (5.4)
**Biology**										
Nt-proBNP, pg/mL: median [iqr]	0	4228.0 [1368.0–10751]	2547.0 [1424.0–4878.0]	2200.0 [1116.0–4962.0]	2585.5 [1364.5–5729.5]	0	1433.0 [498.0–5075.5]	2336.0 [1114.0–3933.0]	2215.0 [1057.0–5276.0]	2256.5 [972.0–4045.0]
Nt-proBNP = 600, pg/mL n (%)	13	56 (88.9)	149 (94.3)	38 (82.6)	243 (91.0)	0	5 (62.5)	51 (92.7)	10 (90.9)	66 (89.2)
Nt-proBNP-eGFR Staging n (%): Stage 1	50	17 (32.7)	71 (53.8)	26 (56.5)	114 (49.6)	1	3 (42.9)	33 (60.0)	7 (63.6)	43 (58.9)
Stage 2		21 (40.4)	37 (28.0)	14 (30.4)	72 (31.3)		4 (57.1)	16 (29.1)	3 (27.3)	23 (31.5)
Stage 3		14 (26.9)	24 (18.2)	6 (13.0)	44 (19.1)		0	6 (10.9)	1 (9.1)	7 (9.6)
Troponin T HS, ng/mL: median [iqr],	0	91.5 [43.5–174.5]	62.0 [43.0–85.0]	72.0 [38.0–106.0]	66.5 [42.0–101.0]	0	42.0 [31.5–93.0]	54.0 [36.0–82.0]	68.0 [35.0–87.0]	54.0 [36.0–84.0]
Hemoglobin, g/dL, mean ± SD	13	12.5 (±1.8)	13.4 (±1.6)	12.7 (±1.6)	13.1 (±1.7)	3	12.4 (±2.1)	13.5 (±1.5)	13.8 (±1.2)	13.4 (±1.6)
Creatinine, μmol/L, mean ± SD	2	136.1 (±96.0)	114.8 (±37.1)	116.5 (±52.2)	119.9 (±58.4)	0	114.9 (±52.3)	111.1 (±36.2)	113.8 (±47.3)	111.9 (±39.2)
eGFR, ml/min/1.73m^2^, mean ± SD	39	60.3 (±29.1)	60.9 (±20.6)	67.7 (±27.9)	62.1 (±24.2)	1	58.8 (±25.0)	64.1 (±15.9)	69.1 (±25.7)	64.4 (±18.4)
**Echocardiography characteristics**										
LVEF, %, mean ± SD	29	53.3 (±9.3)	49.7 (±11.3)	49.5 (±13.0)	50.5 (±11.2)	13	50.7 (±14.8)	50.6 (±9.9)	48.9 (±12.0)	50.4 (±10.6)
IVST, mm, mean ± SD	21	16.5 (±3.7)	17.7 (±3.4)	17.7 (±3.6)	17.4 (±3.5)	5	15.1 (±1.9)	16.9 (±3.1)	17.2 (±2.0)	16.8 (±2.9)
GL Strain, %, mean ± SD	34	11.1 (±4.6)	10.4 (±3.4)	11.6 (±3.8)	10.8 (±3.8)	7	11.8 (±4.4)	10.1 (±3.0)	7.9 (±2.7)	9.9 (±3.2)

SD, standard deviation; BMI, body mass index; CV, cardiovascular; NYHA, New York Heart Association; ICD, implantable cardioverter defibrillator; eGFR, estimated glomerular filtration rate; LVEF, left ventricular ejection fraction; IVST, interventricular septal thickness.

Table 2 highlights the characteristics of the validation prognostic population in 74 patients diagnosed with CA. The mean age was 77.9 ± 9.6, the majority were men (87.8%) and Caucasian (81.1%). The median NT-proBNP and troponin levels were 2256.5 pg/mL [972.0–4045.0] and 54.0 ng/mL [36.0–84.0], respectively (Table 2).

### 3.2. Amylo-AFFECT-QOL item analyses

The Amylo-AFFECT-QOL questionnaire included 33 items scored on a 4-point Likert scale (Table 3). Of the 515 patients included, 296 (57%) completed all items and 219 (43%) had three missing responses at the most (Figure 1). The inter-item Spearman correlation coefficients were all < 0.8, excluding potential redundancy between items. Higher inter-item correlations are seen between “Are you experienced shortness of breath upon exertion?” (Item 1) and “Do you have difficulty climbing stairs because of difficulty breathing?” (Item 3) (rs = 0.71), and between “Do you have tingling and numbness in your fingers and feet?” (Item 9), and “Do you experience tingling?” (Item 12) (rs = 0.68) (Supplementary Table 1).

**TABLE 3:** Description of Amylo-AFFECT’s items scored with a 4-point Lickert scale.

Item	Questions	Dimension associated	N° dimension
1	Are you experienced shortness of breath upon exertion?	Heart failure	1
2	Do you feel like you have difficulty breathing when laying down?	Heart failure	1
3	Do you have difficulty climbing stairs because of difficulty breathing?	Heart failure	1
4	Do you have swollen legs?	Heart failure	1
5	Have you experienced your heart racing? Or, have you ever felt general discomfort?	Vascular dysautonomia	2
6	Have you ever experienced a loss of consciousness?	Vascular dysautonomia	2
7	Do you experience dizziness or discomfort when getting out of bed in the morning, when going from a lying position to a standing position?	Vascular dysautonomia	2
8	Do you have tingling and numbness in your fingers and feet?	Neuropathy	3
9	Have you lost sensitivity in your hands and feet?	Neuropathy	3
10	Do objects fall out of your hands?	Neuropathy	3
11	Do you experience tingling?	Neuropathy	3
12	Do you experience itching?	Neuropathy	3
13	Do you feel cold and is this accompanied by pain?	Neuropathy	3
14	Do you have any problems with, or loss of balance?	Neuropathy	3
15	Do you have difficulty walking?	Heart failure	1
16	Do you have pain or cramps in your legs or arms?	Neuropathy	3
17	Do you have muscle pain?	Neuropathy	3
18	Do you experience tingling in your fingers at night?	Neuropathy	3
19	Do you have diarrhea?	Ear, Gastrointestinal, and urinary dysautonomia	4
20	Are you constipated?	Ear, Gastrointestinal, and urinary dysautonomia	4
21	Do you experience nausea or vomiting?	Vascular dysautonomia	2
22	Are you experiencing a loss of taste? (Ageusia)	Skin or mucosal involvement	5
23	Do you have a dry and coated mouth?	Skin or mucosal involvement	5
24	Has your tongue become swollen? (Macroglossia)	Skin or mucosal involvement	5
25	Do you have difficulty swallowing (tightened throat, need to chew for a very long time, you have a fear of swallowing or ingesting)?	Skin or mucosal involvement	5
26	Has your voice changed? What discomfort does it cause to you?	Skin or mucosal involvement	5
27	Do you have hearing loss?	Ear, Gastrointestinal, and urinary dysautonomia	4
28	Do you have difficulty urinating?	Ear, Gastrointestinal, and urinary dysautonomia	4
29	Do you have difficulty holding your urine?	Ear, Gastrointestinal, and urinary dysautonomia	4
30	Do you have difficulty holding your stool?	Ear, Gastrointestinal, and urinary dysautonomia	4
31	Do you experience sexual dysfunctions?	Ear, Gastrointestinal, and urinary dysautonomia	4
32	Have you had red spots (ecchymosis) around your eyes?	Skin or mucosal involvement	5
33	Have you had red spots (ecchymosis) on your skin?	Skin or mucosal involvement	5

### 3.3. Amylo-AFFECT-QOL’s dimensions structure

The best evaluation seemed to be obtained five dimensions, called: “Heart failure” (1st dimension), “Vascular dysautonomia” (2nd dimension), “Neuropathy” (3rd dimension), “Ear, gastrointestinal, and urinary dysautonomia” (4th dimension), and “Skin or mucosal involvement” (5th dimension) (Table 3). The distribution of items in each dimension, according to the correlation coefficients, is shown in Supplementary Table 2. “Do you have damaged or brittle nails?” (Item 39) was excluded from the analysis because of its lack of consistency. Hence, the scores ranged from 0 to 15 for the 1st dimension, 0–12 for the 2nd dimension, 0–30 for the 3rd dimension, 0–21 for the 4th dimension, and 0–21 for the 5th dimension (Supplementary Table 2).

### 3.4. Questionnaire validation

The developed scale showed good internal consistency (Cronbach’s α = 0.89). Results of the convergent validity study were shown in Table 4. Significant positive correlations were found between global Amylo-AFFECT-QOL and MLHF scores (rs = 0.72) (Table 5). The first dimension (heart failure) of Amylo-AFFECT-QOL was the most correlated with the global score of MLHF (rs = 0.77), especially the physical domain (rs = 0.79). Ear, gastrointestinal, and urinary dysautonomia and skin or mucosal involvement were less correlated with the MLHF global score and dimensions (rs < 0.47).

**TABLE 4:** Results of the confirmatory factorial analysis for the assessment of internal consistency reliability of Amylo-AFFECT questionnaire.

Indicator	Khi2	df	Khi2/df	RMSEA	CFI	NNFI	GFI	AGFI	SRMR	Cronbach’s α
Threshold for a good adjustment			<2	<0.05	>0.9	>0.9	>0.8	>0.8	<0.05	>0.7
Fit measures	864.25	478	1.81	0.04	0.91	0.90	0.91	0.89	0.05	0.89

Df, degree of freedom; RMSEA, root mean square of approximation; CI, confidence interval; NNFI, non-normed fit index; GFI, goodness of fit index; AGFI, adjusted goodness of fit index; SRMR, standardized root mean square residual (evaluation of residuals); AIC, Akaike index criteria.

**TABLE 5:** Comparison between scores of Amylo-AFFECT-QOL and MLHF questionnaires—Convergent validity study.

		MLHF			
		Global score	Physical domain	Emotional domain	Other items
Amylo-AFFECT-QOL	Global score	0.72	0.66	0.62	0.64
	First dimension: Heart failure	0.77	0.79	0.61	0.64
	Second dimension: Vascular dysautonomia	0.54	0.45	0.51	0.49
	Third dimension: Neuropathy	0.5	0.44	0.48	0.44
	Fourth dimension: Gastrointestinal and urinary dysautonomia	0.47	0.39	0.46	0.45
	Fifth dimension: Skin or mucosal involvement	0.46	0.41	0.43	0.45

If the convergence between questionnaires was high, the results of the analyses were closed to 1. The colors correspond to the positive correlations between the global score of Amylo-AFFECT-QOL and MLHF and between each dimension of Amylo-AFFECT-QOL and the domain of MLHF.

### 3.5. Impact of CA and its different types on patient QoL according to the Amylo-AFFECT-QOL score

Patients with a final diagnosis of CA had a global Amylo-AFFECT-QOL score significantly higher than the control group composed by patients with other diagnoses (22.2 ± 13.6 vs. 16.2 ± 13.8, respectively, *p*-value < 0.01). The QoL of patients with CA was more impacted than that of patients with other diagnoses, such as heart failure, ear, gastrointestinal and urinary dysautonomia, and skin or mucosal involvement. The scores for the neuropathy and vascular dysautonomia dimensions were not significantly different (Table 6). According to the Amylo-AFFECT-QOL global results, ATTRv patients’ QoL was more affected than AL patients’ QoL or ATTRwt patients’ QoL. These patients presented with worse QoL, as described by a higher Amylo-AFFECT-QOL score for the 3rd and the 4th dimensions (neuropathy and ear, gastrointestinal and urinary dysautonomia). AL patients had a higher score for the 1st and 5th dimensions (heart failure and skin and mucosal involvement), but its global score was lower than that of ATTRv patients; ATTRwt patients presented a lower score regardless of the dimension.

**TABLE 6:** Amylo-AFFECT-QOL scores of health-related quality of life depending on amyloidosis and its types.

		Overall	Amyloidosis	Other diagnosis	*P*-value ANOVA	AL	ATTRwt	ATTRv with cardiomyopathy with or without neuropathy	*P*-value ANOVA
		*N* = 515	*N* = 419	*N* = 96		*N* = 97	*N* = 246	*N* = 76	
Global score Amylo-AFFECT (0–99)	Missing data	219	182	37	<0.01	50	93	39	0.0051
	Mean ± SD	21.0 ± 13.8	22.2 ± 13.6	16.2 ± 13.8		22.1 ± 13.6	21.2 ± 13.0	26.2 ± 15.6	
Dimension 1 score Heart failure (0–15)	Missing data	36	32	4	0.01	13	13	6	0.0047
	Mean ± SD	6.3 ± 3.8	6.5 ± 3.7	5.5 ± 4.2		7.3 ± 3.8	6.0 ± 3.6	7.0 ± 3.8	
Dimension 2 score Vascular dysautonomia (0–12)	Missing data	25	21	4	0.34	5	13	3	0.0003
	Mean ± SD	1.9 ± 2.3	1.9 ± 2.3	2.1 ± 2.4		2.4 ± 2.5	1.4 ± 2.0	2.4 ± 2.6	
Dimension 3 score Neuropathy (0–30)	Missing data	68	57	11	0.08	13	29	15	0.0071
	Mean ± SD	6.6 ± 6.0	6.9 ± 6.0	5.6 ± 5.7		5.5 ± 5.4	6.8 ± 5.5	8.8 ± 7.8	
Dimension 4 score Gastrointestinal and urinary dysautonomia (0–21)	Missing data	72	60	12	0.03	13	36	11	0.0770
	Mean ± SD	4.1 ± 3.5	4.3 ± 3.4	3.4 ± 3.5		3.8 ± 3.3	4.4 ± 3.3	4.6 ± 3.9	
Dimension 5 score Skin or mucosal involvement (0–21)	Missing data	79	66	13	<0.01	22	27	17	0.0001
	Mean ± SD	2.8 ± 3.2	3.1 ± 3.3	1.7 ± 2.3		4.4 ± 4.0	2.6 ± 2.9	3.1 ± 3.5	

### 3.6. Prognostic value and validation

The results of the regression logistic models are presented in Supplementary Table 3. The median MCO-free survival time (heart transplantation or death) was assessed in all 280 patients. All patients (except those with MCO) had 1 year of follow-up (i.e., Q1, median and Q3 follow-up = 365). Numbers (%) of MCO for the entire cohort were 57 (20.4%) and for AL, ATTRv, and ATTRwt: 27 (42.2%), 12 (24.5%), and 18 (10.8%), respectively.

**FIGURE 2:**
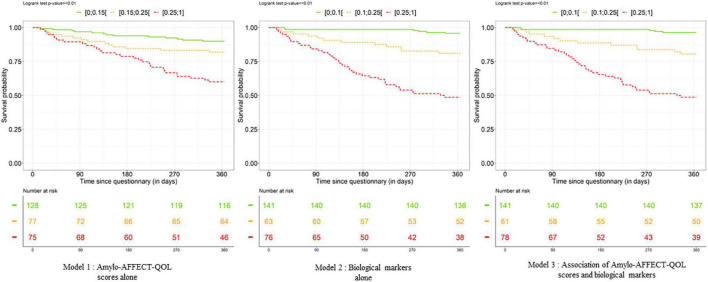
Kaplan–Meier curves describing the link between death or heart transplant and the «calculated» prognostic values (green line represents 50% of patients, orange represents 25% of patients, and red represents 25% of patients) for each model (Amylo-AFFECT-QOL scores alone, biological markers alone, and Amylo-AFFECT-QOL scores and associated biological markers). The lower the prognostic value, the worse the prognosis.

Using the LP, the AUC was smaller for the model using Amylo-AFFECT-QOL dimension scores alone (AUC = 0.73, CI = [0.65–0.80]) than for the model using biological markers alone (AUC = 0.85, CI = [0.79; 0.91]). The AUC values were quite similar for the model using biological markers alone and the model using both inputs. Sensitivity, specificity, negative predictive value (NPV), and positive predictive value (PPV) were calculated for each model for a prognostic value varying from 0.05 to 0.35, as shown in Supplementary Table 4. The best compromise between specificity and sensitivity is given for a prognostic value equal to 0.15 or 0.2. As shown in Figure 2, patients with the worst prognostic value had a greater risk of death or heart transplant after 1 year of follow-up (log-rank < 0.01). The 1-year survival of patients with CA was 61.3% for elevated Amylo-AFFECT-QOL scores and 90.6% for lower scores. The AUC was calculated for VP (Supplementary Table 3). The results confirmed the good performance of our questionnaire as a prognostic value, with an AUC at 0.92 (CI = [0.83; 1.00]).

## 4. Discussion

Improving QoL is an accepted goal in shared decision-making; therefore, it is important to have a self-reported QoL dedicated to patients with CA. This prospective study analyzed the self-report Amylo-AFFECT-QOL questionnaire proposed to a significant sample of French patients suspected of having CA. First, we have demonstrated that the Amylo-AFFECT-QOL questionnaire effectively measures the QoL of CA patients and in a more adapted way than MLHF because it takes into consideration multi-organ damage specific to amyloidosis. Second, we have demonstrated that Amylo-AFFECT-QOL can predict patients’ prognosis.

### 4.1. Structure of QoL evaluation of Amylo-AFFECT-QOL self-reported questionnaire

The need of a specific tool for CA QOL assessment were also identified by other authors (20). There is no single measure or set of measures able to capture the full spectrum of symptoms. Nonetheless, filling many questionnaires would be burdensome for patients, and different measures are partially overlapping and redundant. The Amylo-AFFECT-QOL questionnaire developed by the Reference Center of Henri Mondor Hospital was based on standardized health care HR-QoL questionnaire development with a validation methodology. In contrast with the other generic or organ specific HR-QoL questionnaires used in cases of CA (11–17), this self-reported QoL questionnaire was composed of five dimensions covering the entire spectrum of CA symptoms : heart failure, vascular dysautonomia, neuropathy, ear, gastrointestinal and urinary dysautonomia, and skin or mucosal involvement. The 34 questions of the tool combined with a Likert scale allow a precise and complete assessment of QoL specific to this systemic disease.

### 4.2. Assessment of HR-QoL by Amylo-AFFECT-QOL scoring and correlation with amyloidosis type

In line with the clinical characteristics of amyloidosis, the Amylo-AFFECT-QOL questionnaire demonstrated that CA patients had a worse HR-QoL than patients with other cardiac diseases such as heart failure and patients with gastrointestinal, urinary, and skin dimensions. Among CA patients, patients with ATTRv had a global Amylo-AFFECT-QOL score higher than patients with other types of amyloidosis. This was explained the multisystemic incidence of this type of CA that could be evaluated by the generic characteristics of the Amylo-AFFECT-QOL questionnaire (6). In particular, neuropathy along with gastrointestinal and urinary dysautonomia dimensions played an important role in QoL assessment. Although AL patients presented a QoL reflected by a high score of heart failure and skin and mucosal involvement, their global score was lower than that of ATTRv patients, indicating that their QoL was less impacted. However, AL patients present a very heterogenous phenotype from cardiac involvement only to a large set of symptoms. In line with our results, a recent study using Norfolk QoL-DN showed that ATTRv patients have more impaired QOL than ATTRwt patients owing to dysautonomia (21).

### 4.3. Comparison of the Amylo-AFFECT-QOL questionnaire with the MLHF questionnaire

To date, five different questionnaires have been mostly used, alone or in combination, to evaluate QoL in studies focusing on amyloidosis: SF-36, EQ-5D-3L (10, 22), Northfolk QOL-DN (14), MLHF (17), and KCCQ (9, 23). To validate Amylo-AFFECT-QOL, we compared it to MLHF rather than KCCQ because KCCQ focuses on the impact of dyspnea, a prominent complaint for people with heart failure, in contrast to MLHF, whose 21 questions assess dyspnea but also generic health status and QoL over the period of the previous month (10).

Indeed, global scores to the Amylo-AFFECT-QOL and MLHF questionnaires were correlated due to high correlation for the 1st dimension (Heart Failure issue). Other Amylo-AFFECT-QOL dimensions were less correlated to the MLHF questionnaire. Indeed, screening by Amylo-AFFECT-QOL, which has been specifically designed for CA seems more adapted to evaluation of the multi-organ damage associated with this disease.

### 4.4. The use of Amylo-AFFECT in clinical practice

Amylo-AFFECT may be useful to physicians as well as patients. For the physician, Amylo-AFFECT provides a global vision of the patient that should improve patient care by raising the physician’s awareness on other organs involved than those they are already taking care of (cardiologist might focus on heart). Thus, its use may help physicians to adapte their management and referrals to other organ specialists. For example, in case of AL patients undergoing chemotherapy, it may help hematologists to discriminate amyloidosis-related symptoms from secondary effects of treatment.

Amylo-AFFECT-QOL can also be used to determine patients’ prognosis and adapt their care. CA prognosis is assessed with staging systems relying on levels of the cardiac biomarkers: N-terminal pro b-type natriuretic peptide (NT-proBNP) and troponin (24–27), whose quantification is not accessible in all centers and is expensive, in contrast to a self-reported questionnaire. Moreover, a self-reported questionnaire can evaluate the impact of all potential organ damages of CA on the patient’s QoL thus taking into accounts-non-cardiac prognosis parameters.

We observed that, Amylo-AFFECT had a good prognostic value (AUC of 0.73 (CI = [0.65; 0.80]). It is also a non-invasive, costless and easy-to-use tool in clinical practice.

### 4.5. Study limitation

Our study had some limitations. The results of this study cannot be taken literally owing to the small sample size. Moreover, the follow-up was limited to 1 year. Amylo-AFFECT-QOL global score differences, independent of the severity and number of organs involved, between amyloidosis subtypes may be affected by differences in variables, such as age or sex. We only compared Amylo-AFFECT-QOL to MLHF in this study, and it would be interesting to compare it to other QoL measures.

In this study, we did not assess QoL changes in Amylo-AFFECT during patient’s follow-up and following the start of specific amyloidosis treatment. Serial Amylo-AFFECT evaluation during patient follow-up should help physicians assess progression of the disease and adapt their management. QoL is an important outcome for evaluating the effectiveness of new treatments in patients with CA. Further studies with longer follow-up periods are needed to confirm our results and report the impact of the treatments.

## 5. Conclusion

Amylo-AFFECT-QOL demonstrates good psychometric properties and is useful for quantifying HR-QoL and estimating CA prognosis. It may help improve the overall management of patients with CA. In the future, we need to assess whether Amylo-AFFECT-QOL can capture changes in HR-QoL during disease progression and response to treatment.

## Data availability statement

The raw data supporting the conclusions of this article will be made available by the authors, without undue reservation.

## Ethics statement

The studies involving human participants were reviewed and approved by the Ethics Committee (authorization number #1431858) and informed consent for participation in this research was obtained from all patients. Data were recorded electronically in the Henri Mondor Amyloidosis Network registry, as authorized by the French CNIL (Commission Nationale de l’Informatique et des Libertés). All data were centralized in a secure database. The patients/participants provided their written informed consent to participate in this study.

## Author contributions

MK, MB, and TD contributed to the conception, date curation, investigation, design of the study, supervision, and writing—original draft, review, and editing. TD contributed to the funding acquisition, methodology, project administration, and resources. AB, AG, RG, EP, VM-F, PF, BF, EI, FL, GS, SG, SM, AZ, VA, EA, PL, LH, VP, J-PL, AA, EB, SB, VL, ET, and SO contributed to the data investigation. All authors contributed to the manuscript revision, read, and approved the submitted version.
