# Supplementary material for: Amylo-AFFECT-QOL, a self-reported questionnaire to assess health-related quality of life and to determine the prognosis in cardiac amyloidosis
Source: Front Cardiovasc Med. 2023 Mar 14;10:1124660. doi: 10.3389/fcvm.2023.1124660 (PMC10043221; doi:10.3389/fcvm.2023.1124660)

**Amylo-AFFECT Symptoms and QoL for Amyloidosis Patients**

The purpose of this questionnaire is to evaluate how amylose symptoms affect the patient's quality of life.

**Date:……..…/……….../……..**

**LAST NAME: ……………………………..………. FIRST NAME: ………………….………..……………..…**

**Gender: ………………………………………… Weight:………..… stones/pounds Height: ………… inch/feet**

**Ethnicity: …………………………………**

**Part A: Symptoms**

The following questions refer to symptoms. If you don't have the following symptoms, please check the "No" box. If you show the following symptoms, please check "Yes" for each associated symptom, and fill in when it last occurred: "in the last two years", "more than two years ago”.

**Part B: Quality of life**

The following questions refer to symptoms that occurred in the last 4 weeks and how they have affected your quality of life. To assess how these symptoms affect your quality of life, please select one of the following options for each associated symptom: **0 no discomfort, 1 mild discomfort, 2 moderate discomfort, 3 severe discomfort.**

| ***Amylo-AFFECT***  ***Amyloidosis Symptoms and Quality of life*** | **Part A: Symptoms** | **Part B: Quality of life** |  |
| --- | --- | --- | --- |
|  | ***Have you suffered any of the symptoms in the past?*** | ***Intensity of the symptoms***  ***(over the last 4 weeks)?*** | ***Questions***  ***number*** |
| **Cardiac symptoms** |  |  |  |
| Have you experienced shortness of breath upon exertion? | ◻No or ◻Yes,  **If Yes** : ◻In the last two years ◻ More than two years ago | ◻0  ◻1  ◻ 2  ◻3 | 1 |
| Do you feel like you have difficulty breathing when laying down? | ◻No or ◻Yes,  **If Yes** : ◻In the last two years ◻ More than two years ago | ◻0  ◻1  ◻ 2  ◻3 | 2 |
| Do you have difficulty climbing stairs because of difficulty breathing? | ◻No or ◻Yes,  **If Yes** : ◻In the last two years ◻ More than two years ago | ◻0  ◻1  ◻ 2  ◻3 | 3 |
| Do you have swollen legs? | ◻No or ◻Yes,  **If Yes** : ◻In the last two years ◻ More than two years ago | ◻0 ◻ 1 ◻ 2 ◻ 3 | 4 |
| Have you experienced your heart racing? Or, have you ever felt general discomfort? | ◻No or ◻Yes,  **If Yes** : ◻In the last two years ◻ More than two years ago | ◻0  ◻1  ◻ 2  ◻3 | 5 |
| Have you ever experienced a loss of consciousness? | ◻No or ◻Yes,  **If Yes** : ◻In the last two years ◻ More than two years ago | ◻0 ◻ 1 ◻ 2 ◻ 3 | 6 |
| **Symptoms of orthostatic hypotension** |  |  |  |
| Do you experience dizziness or discomfort when getting out of bed in the morning, when going from a lying position to a standing position? | ◻No or ◻Yes,  **If Yes** : ◻In the last two years ◻ More than two years ago | ◻0 ◻ 1 ◻ 2 ◻ 3 | 7 |
| **Neuromuscular symptoms** |  |  |  |
| Do you have tingling and numbness in your fingers and feet? | ◻No or ◻Yes,  **If Yes** : ◻In the last two years ◻ More than two years ago | ◻0  ◻1  ◻ 2  ◻3 | 8 |
| Have you lost sensitivity in your hands and feet? | ◻No or ◻Yes,  **If Yes** : ◻In the last two years ◻ More than two years ago | ◻0  ◻1  ◻ 2  ◻3 | 9 |
| Do objects fall out of your hands? | ◻No or ◻Yes,  **If Yes** : ◻In the last two years ◻ More than two years ago | ◻0  ◻1  ◻ 2  ◻3 | 10 |
| Do you experience tingling? | ◻No or ◻Yes,  **If Yes** : ◻In the last two years ◻ More than two years ago | ◻0  ◻1  ◻ 2  ◻3 | 11 |
| Do you experience itching? | ◻No or ◻Yes,  **If Yes** : ◻In the last two years ◻ More than two years ago | ◻0  ◻1  ◻ 2  ◻3 | 12 |
| Do you feel cold and is this accompanied by pain? | ◻No or ◻Yes,  **If Yes** : ◻In the last two years ◻ More than two years ago | ◻0  ◻1  ◻ 2  ◻3 | 13 |
| Do you have any problems with, or loss of balance? | ◻No or ◻Yes,  **If Yes** : ◻In the last two years ◻ More than two years ago | ◻0  ◻1  ◻ 2  ◻3 | 14 |
| Do you have difficulty walking? | ◻No or ◻Yes,  **If Yes** : ◻In the last two years ◻ More than two years ago | ◻0  ◻1  ◻ 2  ◻3 | 15 |
| Do you have pain or cramps in your arms or legs? | ◻No or ◻Yes,  **If Yes** : ◻In the last two years ◻ More than two years ago | ◻0  ◻1  ◻ 2  ◻3 | 16 |
| Do you have muscle pain? | ◻No or ◻Yes,  **If Yes** : ◻In the last two years ◻ More than two years ago | ◻0  ◻1  ◻ 2  ◻3 | 17 |
| **Carpal tunnel symptoms** |  |  |  |
| Do you experience tingling in your fingers at night? | ◻No or ◻Yes,  **If Yes** : ◻In the last two years ◻ More than two years ago | ◻0  ◻1  ◻ 2  ◻3 | 18 |
| **Digestive and gastro-intestinal symptoms** |  |  |  |
| Do you have diarrhea? | ◻No or ◻Yes,  **If Yes** : ◻In the last two years ◻ More than two years ago | ◻0  ◻1  ◻ 2  ◻3 | 19 |
| Are you constipated? | ◻No or ◻Yes,  **If Yes** : ◻In the last two years ◻ More than two years ago | ◻0  ◻1  ◻ 2  ◻3 | 20 |
| Do you experience nausea or vomiting? | ◻No or ◻Yes,  **If Yes** : ◻In the last two years ◻ More than two years ago | ◻0  ◻1  ◻ 2  ◻3 | 21 |
| Are you experiencing a loss of taste? (ageusia) | ◻No or ◻Yes,  **If Yes** : ◻In the last two years ◻ More than two years ago | ◻0  ◻1  ◻ 2  ◻3 | 22 |
| Do you have a dry and coated mouth? | ◻No or ◻Yes,  **If Yes** : ◻In the last two years ◻ More than two years ago | ◻0  ◻1  ◻2 ◻3 | 23 |
| Has your tongue become swollen? (macroglossia) | ◻No or ◻Yes,  **If Yes** : ◻In the last two years ◻ More than two years ago | ◻0  ◻1  ◻ 2  ◻3 | 24 |
| Do you have difficulty swallowing?  (Tightened throat, need to chew for a very long time, you have a fear of swallowing or ingesting). | ◻No or ◻Yes,  **If Yes** : ◻In the last two years ◻ More than two years ago | ◻0  ◻1  ◻ 2  ◻3 | 25 |
| Has your voice changed?  What discomfort does this cause you? | ◻No or ◻Yes,  **If Yes** : ◻In the last two years ◻ More than two years ago | ◻Yes ◻ No  ◻0  ◻1  ◻ 2  ◻3 | 26 |
| Do you have hearing loss? | ◻No or ◻Yes,  **If Yes** : ◻In the last two years ◻ More than two years ago | ◻0  ◻1  ◻ 2  ◻3 | 27 |
| **Urinary or genital disorders** |  |  |  |
| Do you have difficulty urinating? | ◻No or ◻Yes,  **If Yes** : ◻In the last two years ◻ More than two years ago | ◻0  ◻1  ◻ 2  ◻3 | 28 |
| Do you have difficulty holding your urine? | ◻No or ◻Yes,  **If Yes** : ◻In the last two years ◻ More than two years ago | ◻0  ◻1  ◻ 2  ◻3 | 29 |
| Do you have difficulty holding your stool? | ◻No or ◻Yes,  **If Yes** : ◻In the last two years ◻ More than two years ago | ◻0  ◻1  ◻ 2  ◻3 | 30 |
| Do you experience sexual dysfunctions? | ◻No or ◻Yes,  **If Yes** : ◻In the last two years ◻ More than two years ago | ◻0  ◻1  ◻ 2  ◻3 | 31 |
| **Impact on the skin** |  |  |  |
| Have you had red spots (ecchymosis) around your eyes? | ◻No or ◻Yes,  **If Yes** : ◻In the last two years ◻ More than two years ago | ◻0  ◻1  ◻ 2  ◻3 | 32 |
| Have you had red spots (ecchymosis) on your skin? | ◻No or ◻Yes,  If Yes : ◻In the last 2 years ◻Before 2 years ago | ◻0  ◻1  ◻ 2  ◻3 | 33 |

**Table of correspondence between the 33 questions and the 5 associated dimensions**


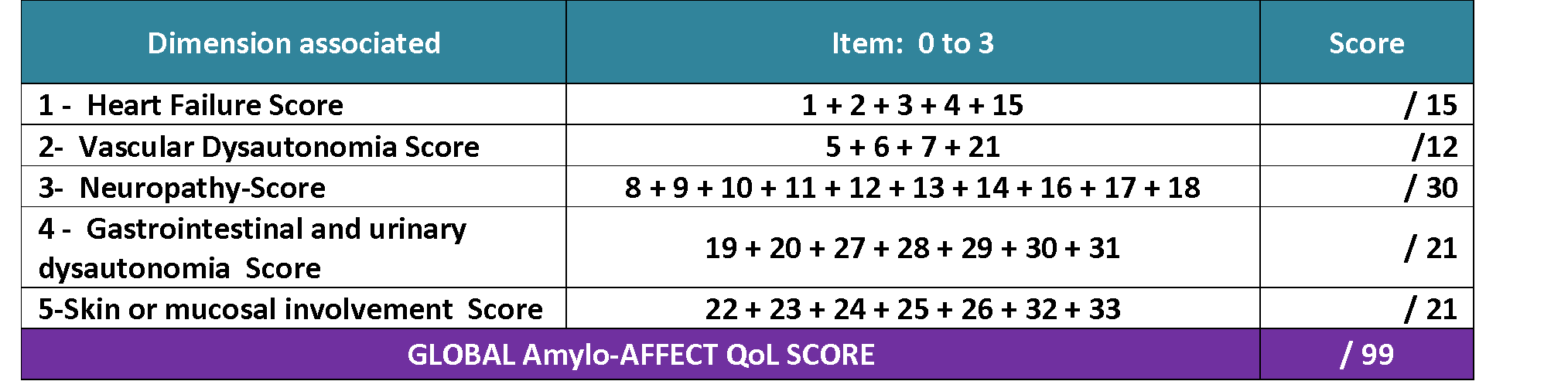

Supplement: Supplementary file 1 [file Table_1.DOCX]
