# Supplementary material for: Amylo-AFFECT-QOL, a self-reported questionnaire to assess health-related quality of life and to determine the prognosis in cardiac amyloidosis
Source: Front Cardiovasc Med. 2023 Mar 14;10:1124660. doi: 10.3389/fcvm.2023.1124660 (PMC10043221; doi:10.3389/fcvm.2023.1124660)
Supplement: Supplementary file 2 [file Table_2.docx]

Supplementary Material 2: Methodology supplement

***Diagnostic criteria of cardiac amyloidosis and subtypes***

The diagnosis of m-TTR amyloidosis was based on identification of a pathogenic TTR gene mutation combined with Congo red staining and anti-TTR antibody labeling of an extracardiac or endomyocardial biopsy specimen. AL was diagnosed based on high monoclonal light-chain levels in serum and/or urine, and on an extracardiac or endomyocardial biopsy showing both Congo red staining and labeling with specific anti-XX or anti-lambda light-chain antibodies. ATTRwt was diagnosed when at least one endomyocardial or extracardiac biopsy specimen exhibited both Congo red staining and labeling with anti-TTR antibodies without a TTR gene mutation; when an extracardiac biopsy was negative and a cardiac biopsy was not ethically acceptable due to advanced age of the patient, the diagnosis of ATTRwt relied on strong scintigraphy uptake of 99mTc-biphosphonate. Cardiac involvement was considered when intraventricular septum thickness (IVST) measured by echocardiography was ≥ 12 mm (without other known cause) and/or cardiac magnetic resonance imaging (MRI) showed diffuse gadolinium enhancement and/or 99mTc-biphosphonate scintigraphy showed strong tracer uptake by the heart. (ref PMID: 27647161)

***“Minnesota Living with Heart Failure” (MLHF)***

The MLHF questionnaire was a self-reported questionnaire for cardiac patients developed by Rector et al. It aimed to assess the health-related quality of life of adult patients with heart failure. It was composed by 21 questions grouped into 3 domains: physical, emotional/psychological, and socio-economics domains. Items were scored on a 6-point Likert scale (from 0 to 5), for the assessment of the degree of disability of each one.

***Translation and Cross-Cultural Validation***

The original Amylo-AFFECT questionnaire was developed in French. Linguistic and cross-cultural adaptation was carried out by a specialized institution (Lionbridge, Dublin, Ireland) to generate Portuguese, English, German, Italian and Spanich, questionnaires version the following methodology was applied: forward and backward translation while accounting for cross-cultural validation and following a nine-step process.

***Internal consistency reliability***

Internal consistency reliability was assessed with a confirmatory factorial analysis.

A theoretical model was defined within an exploratory factorial analysis, during which variables of the Amylo-AFFECT-QOL questionnaire are associated with the 5 dimensions. A confirmatory factorial analysis is used to determine to what extent this theoretical model correctly reproduces the collected data. Several fit indexes are calculated in order to assess good internal consistency:

- the “normalized Chi-square goodness of fit” (chi2/df) is used to evaluate whether sample data is representative of the full population by comparing the gap between the covariance matrix observed on data and that estimated from the theoretical model. A small deviation is obtained with a chi2/df < 2.
- the Goodness of Fit Index (GFI) and the Adjusted Goodness of Fit Index (AGFI) measure the proportion of variance/covariance reproduced by the hypothesized model. A sufficient proportion is obtained with a GFI/AGFI > 0.8.
- the Non-Normed Fit Index (NNFI) and the Comparative Fit Index (CFI) compare the fit of the theoretical model to that of a null model. Questionnaire variables are supposed to be independent. A NFI/CFI > 0.9 indicates that the model of interest has a better fit than the null model.
- The Root Mean Squared Error of Approximation (RMSEA), as indicated, quantifies the root-mean-squared error of the model. Values < 0.05 represent a good fit. This good fit is emphasized when the confidence interval is also below 0.05.
- The Standardized Root Mean Square Residual (SRMR) measures the difference between the residuals of the sample covariance and the hypothesized model. A value < 0.05 indicates a sufficient small difference.
