# Supplementary material for: Amylo-AFFECT-QOL, a self-reported questionnaire to assess health-related quality of life and to determine the prognosis in cardiac amyloidosis
Source: Front Cardiovasc Med. 2023 Mar 14;10:1124660. doi: 10.3389/fcvm.2023.1124660 (PMC10043221; doi:10.3389/fcvm.2023.1124660)
Supplement: Supplementary file 4 [file Table_4.DOCX]

**Supplementary Table 2**: Correlations between items and the five-dimension resulting from the principal composant analysis, and classification of items in each dimension according to these correlations (light grey).

|  |  | **1st factor** | **2^nd^ factor** | **3^rd^ factors** | **4^th^ factor** | **5^th^ factor** |
| --- | --- | --- | --- | --- | --- | --- |
| **Items** | **Range** | **Heart failure** | **Vascular dysautonomia** | **Neuropathy** | **Gastrointestinal and urinary dysautonomia** | **Skin or mucosal involvement** |
| **Item 8** | [0 ; 30] | 0,13 | 0,07 | 0,77 | 0,04 | 0,05 |
| **Item 11** |  | 0,07 | 0,16 | 0,76 | 0,14 | 0,06 |
| **Item 18** |  | -0,01 | 0,05 | 0,67 | 0,00 | 0,10 |
| **Item 9** |  | -0,08 | 0,07 | 0,67 | 0,15 | 0,15 |
| **Item 10** |  | 0,06 | 0,07 | 0,58 | 0,21 | 0,16 |
| **Item 16** |  | 0,20 | 0,06 | 0,52 | 0,10 | 0,09 |
| **Item 17** |  | 0,22 | 0,09 | 0,49 | 0,19 | 0,04 |
| **Item 14** |  | 0,33 | 0,32 | 0,45 | 0,29 | 0,04 |
| **Item 12** |  | 0,15 | 0,01 | 0,38 | 0,30 | 0,26 |
| **Item 13*** |  | 0,10 | 0,10 | 0,35 | 0,08 | 0,36 |
| **Item 25** | [0 ; 21] | 0,11 | 0,09 | 0,03 | 0,16 | 0,66 |
| **Item 24** |  | 0,05 | -0,01 | 0,09 | -0,13 | 0,66 |
| **Item 22** |  | 0,12 | 0,05 | 0,16 | 0,06 | 0,64 |
| **Item 23** |  | 0,16 | 0,07 | 0,25 | 0,19 | 0,57 |
| **Item 26** |  | 0,22 | -0,03 | -0,04 | 0,12 | 0,51 |
| **Item 32** |  | 0,09 | 0,10 | 0,14 | 0,07 | 0,39 |
| **Item 33** |  | 0,19 | -0,21 | 0,15 | 0,26 | 0,28 |
| **Item 3** | [0 ; 15] | 0,76 | 0,25 | 0,07 | 0,06 | 0,19 |
| **Item 1** |  | 0,73 | 0,27 | 0,03 | 0,06 | 0,22 |
| **Item 15** |  | 0,61 | 0,03 | 0,32 | 0,22 | 0,15 |
| **Item 2** |  | 0,48 | 0,26 | 0,07 | -0,05 | 0,33 |
| **Item 4** |  | 0,49 | -0,11 | 0,15 | 0,03 | 0,27 |
| **Item 30** | [0 ; 21] | -0,01 | 0,20 | 0,13 | 0,67 | 0,14 |
| **Item 29** |  | 0,24 | -0,06 | 0,12 | 0,61 | -0,06 |
| **Item 28** |  | 0,01 | 0,03 | 0,11 | 0,59 | 0,12 |
| **Item 19** |  | -0,10 | 0,21 | 0,21 | 0,50 | 0,23 |
| **Item 31** |  | 0,07 | 0,08 | 0,11 | 0,40 | 0,06 |
| **Item 27** |  | 0,41 | -0,17 | 0,24 | 0,37 | -0,13 |
| **Item 20*** |  | 0,17 | -0,01 | 0,29 | 0,23 | 0,24 |
| **Item 6** | [0 ; 12] | 0,02 | 0,69 | 0,09 | 0,15 | -0,03 |
| **Item 5** |  | 0,26 | 0,65 | 0,18 | 0,04 | 0,18 |
| **Item 7** |  | 0,27 | 0,57 | 0,31 | 0,10 | 0,18 |
| **Item 21** |  | -0,01 | 0,35 | 0,10 | 0,16 | 0,43 |

*: Item 14 and 21 were respectively associated to the first and the fourth factor for consistency
