# Supplementary material for: Amylo-AFFECT-QOL, a self-reported questionnaire to assess health-related quality of life and to determine the prognosis in cardiac amyloidosis
Source: Front Cardiovasc Med. 2023 Mar 14;10:1124660. doi: 10.3389/fcvm.2023.1124660 (PMC10043221; doi:10.3389/fcvm.2023.1124660)
Supplement: Supplementary file 5 [file Table_5.DOCX]

**Supplementary Table 3**: Parameters resulting from logistic regression models and calculation of AUC for the learning and validation populations.

|  |  | Dimensions scores alone | Biological markers scores alone | Dimensions and biological markers scores associated |
| --- | --- | --- | --- | --- |
| β | Intercept | -2.603130 | -11.2628 | -11.003810 |
|  | log (NT-pro BNP) |  | 1.0531 | 0.982796 |
|  | Troponin |  | 0.0117 | 0.011563 |
|  | 1st dimension score | 0.154676 |  | 0.051330 |
|  | 2nd dimension score | 0.030135 |  | 0.037984 |
|  | 3rd dimension score neuro | -0.056195 |  | 0.013279 |
|  | 4th dimension score | -0.020035 |  | -0.082005 |
|  | 5th dimension score skin | 0.131401 |  | 0.038880 |
| AUC | Learning population | 0.73 [0.65;0.80] | 0.85 [0.79;0.91] | 0.86 [0.81;0.92] |
|  | Validation population | 0.92 [0.83;1.00] | 0.72 [0.50;0,95] | 0.78 [0.58;0.98] |

The prognostic value is calculated with the following formula:

$$Pr\left( Output/X_{i} \right)=\frac{\exp\left( \sum_{i} \beta_{i}X_{i} \right)}{1+\exp\left( \sum_{i} \beta_{i}X_{i} \right)}$$
