# Supplementary material for: Amylo-AFFECT-QOL, a self-reported questionnaire to assess health-related quality of life and to determine the prognosis in cardiac amyloidosis
Source: Front Cardiovasc Med. 2023 Mar 14;10:1124660. doi: 10.3389/fcvm.2023.1124660 (PMC10043221; doi:10.3389/fcvm.2023.1124660)
Supplement: Supplementary file 6 [file Table_6.DOCX]

**Supplementary Table 4** : Results of sensitivity, specificity, NPV, and PPV according to prognostic value.

|  |  | Amylo-AFFECT  dimensions scores alone | | Biological markers alone | | Amylo-AFFECT  dimensions and biological markers scores associated | |
| --- | --- | --- | --- | --- | --- | --- | --- |
| Prognostic value | Parameter | Value | CI | Value | CI | Value | CI |
| 0.05 | Accuracy | 0.21 | [0.16;0.26] | 0.47 | [0.41;0.53] | 0.52 | [0.46;0.57] |
|  | Sensitivity | 1.00 | [1.00;1.00] | 0.95 | [0.89;1.00] | 0.98 | [0.95;1.00] |
|  | Specificity | 0.01 | [0.00;0.02] | 0.35 | [0.29;0.41] | 0.40 | [0.33;0.46] |
|  | NPV | 1.00 | [1.00;1.00] | 0.96 | [0.92;1.00] | 0.99 | [0.97;1.00] |
|  | PPV | 0.20 | [0.16;0.25] | 0.27 | [0.21;0.33] | 0.29 | [0.23;0.36] |
| 0.10 | Accuracy | 0.41 | [0.35;0.47] | 0.66 | [0.61;0.72] | 0.67 | [0.61;0.73] |
|  | Sensitivity | 0.96 | [0.92;1.00] | 0.89 | [0.82;0.97] | 0.91 | [0.84;0.99] |
|  | Specificity | 0.27 | [0.21;0.33] | 0.61 | [0.54;0.67] | 0.61 | [0.54;0.67] |
|  | NPV | 0.97 | [0.92;1.00] | 0.96 | [0.92;0.99] | 0.96 | [0.93;1.00] |
|  | PPV | 0.25 | [0.19;0.31] | 0.37 | [0.29;0.45] | 0.37 | [0.29;0.45] |
| 0.15 | Accuracy | 0.56 | [0.51;0.62] | 0.71 | [0.65;0.76] | 0.74 | [0.68;0.79] |
|  | Sensitivity | 0.77 | [0.66;0.88] | 0.79 | [0.68;0.90] | 0.81 | [0.71;0.92] |
|  | Specificity | 0.51 | [0.45;0.58] | 0.69 | [0.63;0.75] | 0.72 | [0.66;0.78] |
|  | NPV | 0.90 | [0.85;0.95] | 0.93 | [0.89;0.97] | 0.94 | [0.90;0.97] |
|  | PPV | 0.29 | [0.22;0.36] | 0.39 | [0.30;0.48] | 0.42 | [0.33;0.52] |
| 0.175 | Accuracy | 0.66 | [0.60;0.71] | 0.75 | [0.70;0.80] | 0.76 | [0.71;0.81] |
|  | Sensitivity | 0.72 | [0.60;0.84] | 0.79 | [0.68;0.90] | 0.77 | [0.66;0.88] |
|  | Specificity | 0.64 | [0.58;0.70] | 0.74 | [0.68;0.79] | 0.76 | [0.70;0.82] |
|  | NPV | 0.90 | [0.85;0.95] | 0.93 | [0.89;0.97] | 0.93 | [0.89;0.97] |
|  | PPV | 0.34 | [0.25;0.42] | 0.43 | [0.34;0.53] | 0.45 | [0.35;0.55] |
| 0.20 | Accuracy | 0.69 | [0.63;0.74] | 0.77 | [0.72;0.82] | 0.78 | [0.73;0.83] |
|  | Sensitivity | 0.62 | [0.49;0.75] | 0.75 | [0.64;0.87] | 0.75 | [0.64;0.87] |
|  | Specificity | 0.71 | [0.65;0.77] | 0.77 | [0.72;0.83] | 0.78 | [0.73;0.84] |
|  | NPV | 0.88 | [0.83;0.93] | 0.92 | [0.89;0.96] | 0.93 | [0.89;0.96] |
|  | PPV | 0.35 | [0.26;0.45] | 0.46 | [0.36;0.56] | 0.47 | [0.37;0.58] |
| 0.25 | Accuracy | 0.74 | [0.69;0.79] | 0.80 | [0.76;0.85] | 0.80 | [0.75;0.85] |
|  | Sensitivity | 0.53 | [0.40;0.66] | 0.68 | [0.56;0.80] | 0.70 | [0.58;0.82] |
|  | Specificity | 0.79 | [0.74;0.85] | 0.83 | [0.79;0.88] | 0.83 | [0.78;0.88] |
|  | NPV | 0.87 | [0.82;0.92] | 0.91 | [0.87;0.95] | 0.92 | [0.88;0.95] |
|  | PPV | 0.40 | [0.29;0.51] | 0.51 | [0.40;0.63] | 0.51 | [0.40;0.62] |
| 0.30 | Accuracy | 0.78 | [0.73;0.82] | 0.82 | [0.78;0.87] | 0.83 | [0.79;0.87] |
|  | Sensitivity | 0.42 | [0.29;0.55] | 0.61 | [0.49;0.74] | 0.65 | [0.53;0.77] |
|  | Specificity | 0.87 | [0.82;0.91] | 0.88 | [0.84;0.92] | 0.88 | [0.83;0.92] |
|  | NPV | 0.85 | [0.81;0.90] | 0.90 | [0.86;0.94] | 0.91 | [0.87;0.95] |
|  | PPV | 0.44 | [0.31;0.58] | 0.56 | [0.44;0.69] | 0.57 | [0.45;0.69] |
| 0.35 | Accuracy | 0.79 | [0.75;0.84] | 0.84 | [0.79;0.88] | 0.86 | [0.82;0.90] |
|  | Sensitivity | 0.35 | [0.21;0.48] | 0.56 | [0.43;0.69] | 0.63 | [0.51;0.76] |
|  | Specificity | 0.91 | [0.87;0.95] | 0.91 | [0.87;0.94] | 0.92 | [0.88;0.96] |
|  | NPV | 0.84 | [0.80;0.89] | 0.89 | [0.85;0.93] | 0.91 | [0.87;0.94] |
|  | PPV | 0.49 | [0.33;0.65] | 0.60 | [0.47;0.74 | 0.67 | [0.54;0.80] |
